# Flu and Covid-19: is there adherence to vaccination in target groups? Domitilla Marconi

**DOI:** 10.1093/eurpub/ckac130.227

**Published:** 2022-10-25

**Authors:** D Marconi, L Botarelli, M Gennari, A Cartocci, N Vigiani, N Nante, G Messina

**Affiliations:** 1 Post Graduate School of Public Health, University of Siena, Siena, Italy; 2 Department of Medical Biotechnology, University of Siena, Siena, Italy; 3 UOC Food and Nutrition Hygiene-East Area, Azienda USL Toscana Sud-Est, Arezzo, Italy; 4 Department of Molecular and Developmental Medicine, University of Siena, Siena, Italy

## Abstract

**Background:**

In Italy, the flu vaccine is recommended and free for target groups (adults≥60 years old, fragile people, healthcare workers, pregnant women). During the 2020/2021 flu season, an increased vaccination coverage (+6,9%) was observed compared to the previous season, also due to the Covid-19 pandemic. We aimed to investigate how strong the adherence to the flu vaccine was by the vulnerable groups and assess if the Covid-19 vaccination campaign may have influenced the rate of flu vaccines.

**Methods:**

At the beginning of autumn 2021 we conducted an online survey among the population of Tuscany. We collected data on demographics, health status (pregnancy, vulnerable), flu and COVID-19 vaccinations coverage and health information sources. In addition, we performed a descriptive and a risk factors analysis to assess correlation between our variables with R v 4.0.0. Significance level was set at p < 0.05.

**Results:**

Among 408 participants, 248 (61%) belong to a vulnerable group and are recommended to receive the flu vaccine, 229 (56%) usually get the flu vaccine, 386 (95%) got the Covid-19 vaccine, 267 (65%) choose and trust the general practitioner (GP) as their health information source. There is a statistically significant association between being part of a vulnerable group and getting the seasonal flu vaccine (OR 6.63 95% CI 4.26-10.3 p < 0.001). In addition, getting the Covid-19 vaccine increases the likelihood of receiving the flu vaccine (2.90 95% CI 1.16-7.28 p = 0.018). Moreover, participants who trust their GP as their health information source (OR 1.63 CI 1.08-2.46 p = 0.019) are more likely to receive the flu vaccine; other information sources (TV, newspaper, social media) are not associated with the flu vaccine.

**Conclusions:**

Our research shows that vulnerable groups get vaccinated against the flu. The increase in flu vaccine coverage may be due to the COVID-19 vaccines campaign. GPs play a crucial role in the health promotion, prevention and health literacy of patients.

**Key messages:**

- GP plays a crucial role in the prevention and health literacy of patients.
- COVID-19 vaccines campaign may have positively influenced the flu vaccination campaign. However, we must continue to maintain the coverage we have managed to achieve in the future.

